# Colloidal Processing of Y_0.08_Zr_0.92_O_2_/La_0.80_Sr_0.20_MnO_3_ Semi-Cells Using a Sr-Doped Lanthanum Manganite Synthesized by a Citrate Route

**DOI:** 10.3390/ma14247831

**Published:** 2021-12-17

**Authors:** Paloma Recio, Carmen Alcázar, Rodrigo Moreno

**Affiliations:** Instituto de Cerámica y Vidrio, CSIC, Kelsen 5, E-28049 Madrid, Spain; precio@icv.csic.es (P.R.); carmen.alcazar@icv.csic.es (C.A.)

**Keywords:** lanthanum manganite, synthesis, Pechini, suspensions, dip-coating, processing, solid oxide fuel cells

## Abstract

In this paper, the interface between yttria stabilized zirconia (Y_0.08_Zr_0.92_O_2_, YSZ) electrolyte and Sr-doped lanthanum manganite (La_0.80_Sr_0.20_MnO_3_, LSM) cathode for solid oxide fuel cells (SOFCs) is studied. For such a purpose, the combination of a suitable synthesis route for obtaining fine powders and simple aqueous colloidal shaping routes is proposed. The synthesis of nanosized particles of La_0.80_Sr_0.20_MnO_3_ by a citrate route and their full characterization, including the colloidal stability and the densification and phase development determined by X-ray diffraction and electron microscopy at different temperatures, is reported. In a second step, YSZ tapes were obtained by aqueous tape casting and used as substrates for the preparation of LSM coatings by dip-coating using aqueous slurries. YSZ tapes were used either in the green state or after a pre-sintering treatment. Co-sintering at 1350 °C led to a sharp interface with excellent adhesion, also achieved when coating pre-sintered tapes. In both cases, the substrates are dense and the coatings are porous, with thicknesses of 85 and 60 μm for green and pre-sintered tapes, respectively. No diffusion of Zr and Y occurs at the LSM layer, but some diffusion of La and Mn towards the YSZ layer takes place.

## 1. Introduction

La_1−x_Sr_x_MnO_3_ (LSM) oxides have received increasing attention in the last years thanks to their complex chemistry that allows the synthesis of a broad number of compositions, the excellent electrical and magnetic properties, and the good mechanical behavior, which make them suitable for a variety of applications in several technological sectors such as medicine, energy, chemistry, and magnetism [1,2,3,4]. The most extended applications are related to their high catalytic activity in automotive exhaust control and for the elimination of CO pollution [5,6], the ferromagnetic properties of manganite nanoparticles that exhibit very low Curie temperatures [7], and the production of cathodes for solid oxide fuel cells (SOFCs) [8,9,10]. Among the cathode materials for SOFCs, manganites are probably the most popular because of their high electronic conductivity and the chemical stability and compatibility with the most frequently used electrolyte (zirconia stabilized with 8 mol% yttria, YSZ). Therefore, the development of different routes for the synthesis of manganites with tailored compositions has attracted the interest of the scientific community.

Physical methods such as mechanosynthesis [11] and solid-state reactions [12] have been largely used for the synthesis of manganites, but in spite of the advantages of the lower cost of oxide precursors and simplicity, the physical methods require the use of high temperatures and long processing times and lead to some contamination the former and coarse particle size the last. Consequently, physical routes have been progressively replaced by wet chemical methods that make possible the synthesis of many different compositions, changing the relative ratio of the forming cations and allowing achieving high purity powders with a lower particle size in short processing times. Among the wet chemical routes for the synthesis of LSM, the most extensively used include reverse micellization [13], sol-gel [14,15], co-precipitation [16], combustion [17,18], spray-pyrolysis [19], microwave-assisted low temperature synthesis [20], and citrate gel route [3,21]. This method was developed by Pechini [22] and utilizes citric acid to form chelates of the metal ions and ethylene glycol as solvent for the polymerization leading to a polyester-type resin. A cost-effective process known as chemical pyrophoric reaction has been also used for the synthesis of Fe-doped LSM-based powders using La_2_O_3_ [23].

The effort in producing nanosized powders with controlled characteristics must be accompanied by an efficient processing to confer the desired shape to the component, which is of particular importance in the case of complex structures such as those of SOFCs, comprising layers formed by different materials with specific properties, and different porosity and thermal expansion coefficients. Therefore, the selection of adequate shaping techniques is critical for the production of such layered structures. Several shaping techniques have been used for the manufacture of porous LSM electrodes, including pressing [22], extrusion [24], screen printing [25], dip coating [26], and tape casting [27,28]. Another way to improve the efficiency of the cell is maximizing the contact area between electrode and electrolyte, for instance, by pulsed laser machining of YSZ and subsequent dip coating of an LSM/YSZ composite [29].

Among the different processing strategies, colloidal methods have grown considerably, in particular with the use of aqueous suspensions owing to health, environmental, and economic considerations [30]. The integration of different colloidal forming procedures to build-up a semi-cell with YSZ electrolyte and LSM as a cathode controlling the colloidal and rheological properties has received less attention than required. Only few works have reported measurements of the isoelectric point of LSM and the rheological properties of LSM suspensions.

The objective of this work is twofold: on one hand, to optimize the synthesis of fine LSM particles by a citrate gel route as well as the sintering conditions and evolution of phases with temperature; on the other hand, to produce a semi-cell YSZ-LSM by dip-coating an aqueous slurry of the synthesized LSM powder on green or pre-sintered YSZ substrates manufactured by aqueous tape casting.

## 2. Materials and Methods

### 2.1. Synthesis and Characterization of LSM Powders

A strontium-doped lanthanum manganite powder with the composition La_0.80_Sr_0.20_MnO_3_ was synthesized by a Pechini route using the corresponding metal nitrates, La(NO_3_)_3_∙6H_2_O, Mn(NO_3_)_2_∙4H_2_O, and Sr(NO_3_)_2_. The Pechini process is based on the poly-esterification between a quelate (carboxylic acid—metal) and a poly-hydroxylated alcohol. Citric acid (C₆H₈O₇) and ethylene glycol (C_2_H_6_O_2_) were used as the carboxylic acid and the poly-hydroxylated alcohol, respectively. All reagents were purchased from Merck (Darmstadt, Germany).

The chemical reagents used in this work were of analytical grade and used as received without further purification. All aqueous solutions were prepared with deionized water with a resistivity of >18 MΩ cm, produced by a Milli-Q Plus pure water generating system from Millipore (Bedford, MA, USA).

For comparison purposes, a commercial powder of the same composition was also used (LSM80, Inframat Advanced Materials, Willington, DE, USA). According to the supplier, this powder has a specific surface area of 2.8 m^2^/g, an average particle size of 0.25 μm, and a density of 6.30 g/cm^3^.

**Figure 1:**
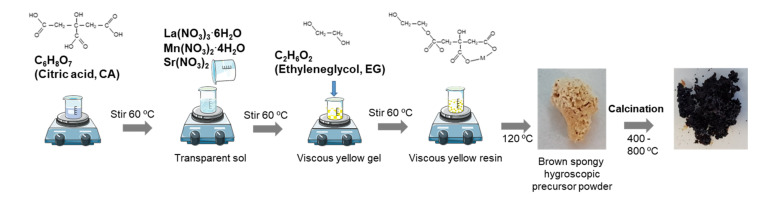
Schematic representation of the complete synthesis procedure.

The complete synthesis procedure is graphically represented in Figure 1. Stoichiometric amounts of starting metal nitrates La(NO_3_)_3_∙6H_2_O, Mn(NO_3_)_2_∙4H_2_O, and Sr(NO_3_)_2_ were dissolved in 100 mL of water and stirred for about 30 min and subsequently added dropwise into a citric acid (CA) solution to promote the chelation of the metal cations. The CA/total oxides molar ratio (CA/MO) was fixed to 2:1. The mixtures were maintained in a glycerin bath at 60 °C with constant stirring for about 1 h. The addition of the metal ions M (with M = Mn, La, Sr) solutions to the citrate heated at 60 °C allows the formation of poly-basic acid chelates. These citrate ion complexes are quite stable thanks to the strong coordination of the metal ion with the citrate ion including one hydroxyl and two carboxyl groups, which lead to a transparent solution of the mixture without precipitates. After 1 h agitation, ethylene glycol (EG) was added to the precursor solution with a CA/EG molar ratio of 1:5 and heated in a glycerin bath at 90 °C until gelation was completed. The addition of EG provokes the formation of an organic ester with the acid chelates, and when these solutions of metal ions in the organic matrix are heated, the excess of solvent is removed and an intermediate resin with the cations uniformly distributed throughout is formed. In the picture, the molecular structure for La-quelate is shown, but similar quelates of Mn and Sr form and all them combine into a structural network. The as-prepared yellowish resin was then dried in a stove at 120 °C for 15 h and a brown, spongy hygroscopic powder was obtained. In a first step, the fine LSM powders were obtained by calcining the dried gel at 400 °C, 600 °C, and 800 °C for 4 h. The powders treated in this way were thermally characterized in order to study the phase’s evolution. After optimization, the powders were treated at 800 °C/4 h to obtain the desired phases.

The precursor powders obtained at 800 °C were compacted by uniaxial pressing with a pressure of 200 MPa to produce discs with ~ 3 mm in height. The green discs were subjected to different thermal treatments ranging from 1000 to 1400 °C until the desired crystallographic phases were formed. These treatments were fixed on the basis of thermal analysis and dynamic sintering studies.

The particle size distribution of the synthesized powders was measured by dynamic light scattering using a NanoZS (Malvern Instruments, Malvern, UK) apparatus. Specific surface areas of the powders were measured by the BET method using nitrogen as an adsorbate (Quantachrome MS-16 model, Syosset, NY, USA). The stability in water of both the commercial and the synthesized and thermally treated powders was determined through zeta potential measurements as a function of pH by the laser Doppler electrophoresis technique using the same equipment as for particle size. Suspensions were prepared in deionized water to a concentration of 0.1 g/L using 10^−2^ M KCl as inert electrolyte. pH was adjusted with HCl and KOH.

The crystalline phases of the powders before and after thermal treatments were determined by X-ray diffraction with a D8 Advance (Bruker, Karlsruhe, Germany) diffractometer using Cu Kα radiation CuKα, λ = 1.5405 A. The measurements were performed in the range of 10°–70° 2θ for powders and 20°–120° for sintered specimens, and the step size and time of reading were 0.05° and 1.5 s, respectively. The obtained XRD patterns were analyzed using the diffraction files of La_0.80_Sr_0.20_MnO_3_, PDF 01-073-6750; La(OH)_3_, PDF 00-036-1481; La_2_O_3_, PDF 00-05-0602; and Mn_3_O_4_, PDF 00-024-0734. All files were collected at the ICDD© databank (JCPDS-The International Centre for Diffraction Data©, Newton Square, Delaware County, PA, USA). The crystallite size of the LSM powders was calculated from the XRD spectra calculating the full width half maximum (FWHM) of most intensity reflection (110) using the Debye–Scherrer equation.

Dynamic sintering studies were performed by constant heating rate experiments with a differential dilatometer (Setsys 16/18, Setaram, Caluire, France) up to 1550 °C and heating/cooling rates of 5 °C/min using pressed cylindrical samples. Isothermal sintering experiments were performed at temperatures ranging from 1000 to 1400 °C in intervals of 100 °C, with heating and cooling rates of 5 °C/min, in a laboratory furnace. The holding time was 4 h. The thermal behavior and the weight loss as a function of temperature were determined with a thermal differential and thermogravimetry analyzer (DTA–TG, STA 409, Netzsch, Selb, Germany) up to 1400 °C with a heating rate of 5 °C/min. The density of sintered bodies was measured by the Archimedes method using deionized water.

The microstructure of the powders and the compacts treated at different temperatures was observed by field emission gun-scanning electron microscopy with energy dispersive X-ray (FE-SEM-EDX, Hitachi S-4700 type I, Tokyo, Japan) microanalysis. Powders were also observed by high-resolution transmission electron microscopy (HR-TEM) on a JEM-2100F (JEOL, Tokyo, Japan) microscope working at 200 keV.

### 2.2. Shaping of LSM Coatings onto YSZ Tapes

The synthesized LSM powder performance was evaluated by preparing LSM coatings onto ZrO_2_ doped with 8 mol% Y_2_O_3_ (Tosoh TZ-8YS, Tokyo, Japan) substrates prepared in the laboratory by tape casting in water according to the procedure described elsewhere [31]. In summary, suspensions were prepared to a solids content of 45 vol.%, adding 0.5 wt% of polyacrylic-based polyelectrolyte (Duramax D3005, Rohm & Haas, Collegeville, PA, USA) as a deflocculant and 10 wt% of an acrylic latex emulsion (Duramax B-1000, Rohm &Haas, Collegeville, PA, USA) as a binder. The green tapes were cut to obtain rectangular samples of 3.0 cm × 1.5 cm. LSM coatings were performed in two ways, firstly using YSZ green tapes as substrates and co-sintering, and secondly using YSZ tapes pre-sintered at 1300 °C. LSM dipping suspensions were prepared in water by mechanical agitation with helices to a solids loading of 15 vol.% using the same deflocculant used for YSZ tapes (PAA) at a concentration of 2 wt% on a dry solids basis. Dipping tests were carried out with a lift using immersion/withdrawal rates ranging from 0.4 to 0.8 cm/s. Coated samples were sintered at 1350 °C/2 h at heating/cooling rates of 5 °C/min.

Coated specimens were characterized by X-ray diffraction, taking powder scratched from the coating. Microstructural observations of sintered coatings were done on the surfaces and the cross-sections of the coated materials. To highlight the grain boundaries, samples were chemically etched with HF. Elemental phase composition was determined with an energy dispersive spectroscope (EDS) detector, measuring an EDS line scan along the LSM/YSZ interface.

## 3. Results

### 3.1. Synthesis and Characterization of LSM Powders

**Figure 2:**
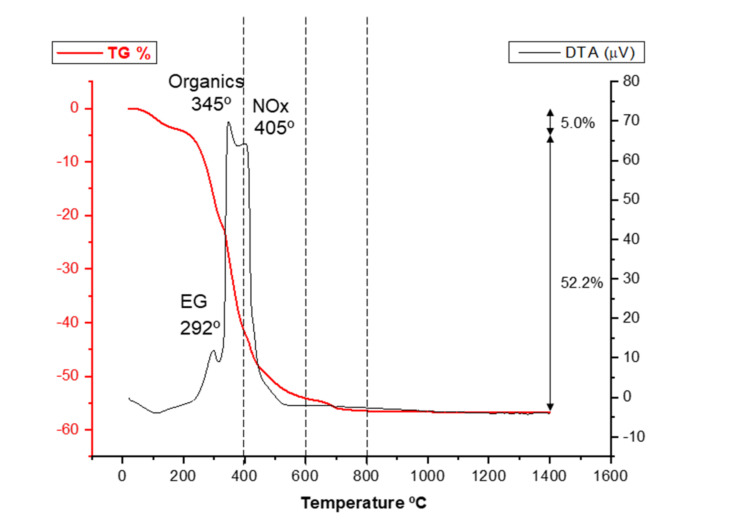
Differential thermal analysis (DTA) and thermogravimetry (TG) of the as-synthesized LSM powder.

Once the LSM precursor powder has been synthesized, it must be calcined to form the oxide phases. However, the calcination temperature determines the development of new phases as well as the retention of undesired phases, such as the original nitrates or the hydroxides formed at early temperatures. In order to select the adequate calcination treatment for obtaining the desired phases, a first thermal analysis was performed on the LSM precursor. Figure 2 shows the results of the differential thermal analysis (DTA) and thermogravimetry (TG). In the TG curve, there is a first weight loss between room temperature and 200 °C related to evaporation of water adsorbed to the quelates and the weight loss in this region is 5.0%. The largest weight loss (48.7%) occurs in a second region at temperatures ranging from 200 to 500 °C, which is attributed to the elimination of CO_2_ and NO_x_ gases originated by ethylene glycol evaporation, the decomposition of the excess of citrate, and the break of the complex metal–citrate. Finally, there is a third region at temperatures of 600–700 °C corresponding to the crystallization of the perovskite phase in which a weight loss of 3.5% is recorded. The differential thermal analysis reveals an endothermic effect at 100 °C associated with desorption of physically adsorbed water. At intermediate temperatures, three exothermic effects with their maximum peaks centered at temperatures of 292, 345, and 405 °C can be detected, which are related to the destruction of the organic compounds involved in the Pechini synthesis route. The temperature and width of these exothermic events can change depending on the ratio between the concentration of nitrates and the total concentration of metal ions used for the synthesis, as reported in the literature [6,32]. Finally, at temperatures ranging from 500 to 700 °C, neither exothermic nor endothermic effects are recorded in the DTA curve. The fact that, in this zone, a weight loss of 3.5% is registered seems to indicate that the formation of the perovskite phase occurs through an intermediate amorphous phase of oxyhydroxy carbonate type, as reported elsewhere [33].

**Figure 3:**
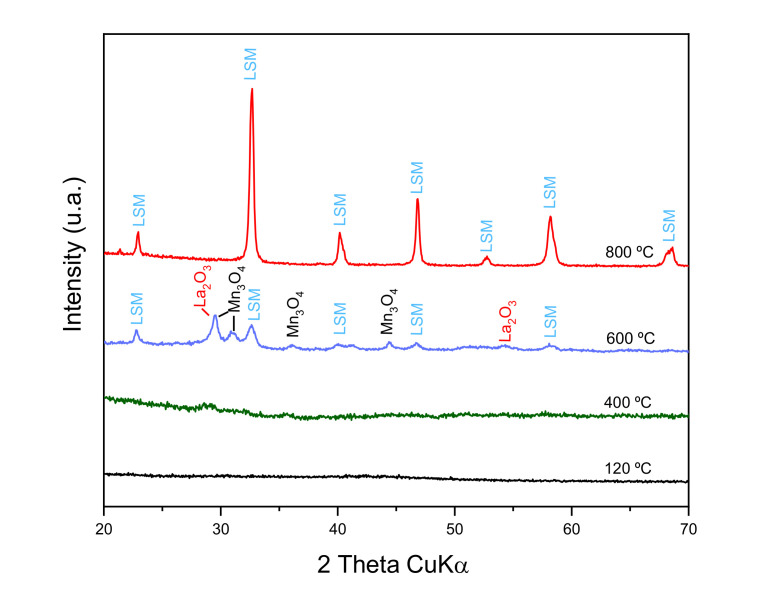
X-ray diffraction patterns of the LSM powder synthesized by citrate route treated at different temperatures.

According to the DTA/TG analysis, different portions of the as-synthesized powder were subjected to thermal treatments at 400, 600, and 800 °C. The resulting calcined powders were analyzed by XRD in order to study the evolution of phases with temperature. Figure 3 shows the obtained XRD patterns. At a low temperature, 400 °C and less, there are no diffraction peaks, so no crystalline phases are still formed. At 600 °C, small diffraction signals are observed, showing the incipient formation of LSM, but with large concentrations of the former oxides (La, Mn) still remaining. At 800 °C, high intensity, narrow diffraction peaks of LSM as unique phase are detected, without secondary phases. This demonstrates the importance of the weight loss detected at around 700 °C in the TG curve (Figure 2).

**Figure 4:**
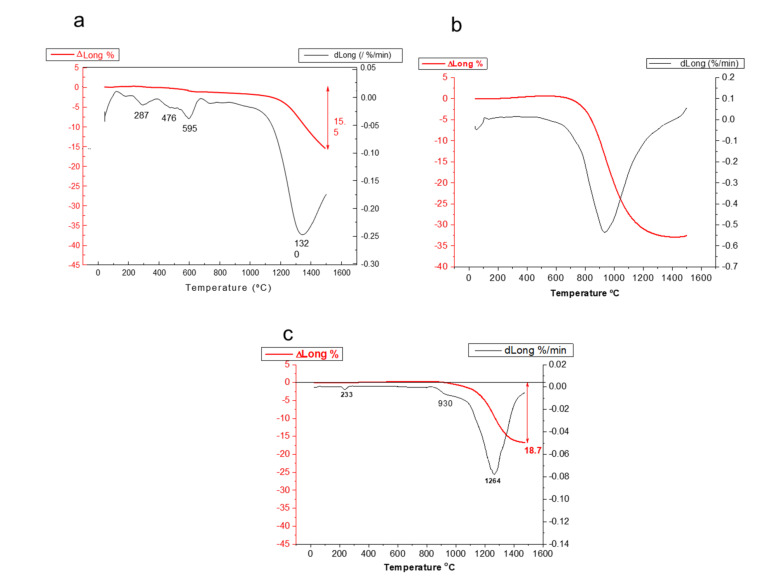
Dynamic sintering curves for synthesized LSM powders treated at 400 °C (**a**) and 800 °C (**b**), compared with the behavior of the commercial powder (**c**).

The sinterability of the calcined powders was evaluated by dynamic sintering studies of the powders compacted by uniaxial pressing at 60 MPa. The dilatometric curves of samples calcined at 400 and 800 °C are plotted in Figure 4a,b, respectively. The dilatometry of the commercial LSM powders is shown in Figure 4c for comparison purposes. For the sample treated at 400 °C, the shrinkage starts to occur at 1200 °C and, at 1550 °C, the total shrinkage is only 15.5%, very far from the expected shrinkage for a dense compact. The derivative curve shows several peaks at 290, 475, and 595 °C and a minimum at 1350 °C that corresponds to the maximum sintering rate. For the powder calcined at 800 °C, the sintering curve is quite different. The shrinkage starts at above 800 °C, with continuous progression until 1400 °C, where a plateau is reached, thus suggesting that a good densification level is achieved, with a maximum shrinkage value of 33%, much higher than that observed in the powder calcined at 400 °C. The maximum densification rate in this case takes place at 930 °C. This is due to the densification of the LSM, whereas in the previous case, the presence of the hydroxides and the incomplete formation of LSM make sintering difficult. Figure 4c shows the behavior of the commercial powder. It can be seen that the shrinkage starts at 1000 °C and, at 1500 °C, the maximum shrinkage is 19.5%. The derivative curve shows a first small minimum at 930 °C and a big minimum corresponding to the maximum densification rate at 1264 °C. The commercial powder is calcined for the commercial distribution so the amount of organic phases retained in the powder is much lower than in the as-synthesized powder obtained by the Pechini route.

Considering all previous data, it is clear that the desired LSM phase is obtained when the powder is calcined at 800 °C, and this powder was thus characterized and selected for further studies.

**Figure 5:**
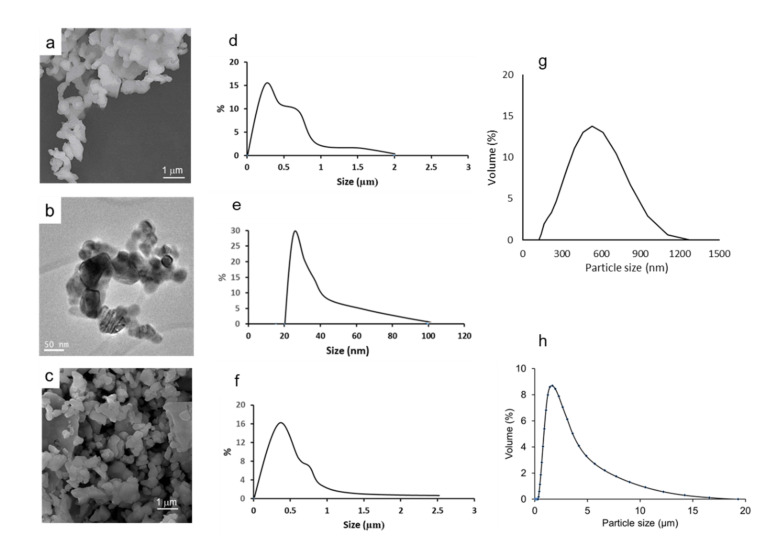
Morphology of the synthesized LSM powder observed by SEM (**a**) and TEM (**b**) and SEM morphology of the commercial powder (**c**), particle size distribution evaluated by direct measurement in the corresponding pictures (**d**,**e**) for SEM and TEM, respectively, of the synthesized powder treated at 800 °C and (**f**) for the SEM picture of commercial powder. The particle size distribution measured by laser diffraction for the synthesized powder treated at 800 °C (**g**) and the commercial powder (**h**) are also shown.

In the first place, the morphology and stability of the calcined powders were studied. The microstructure of the powders observed by SEM and TEM can be seen in Figure 5a,b, respectively. From the electron microscopy images, a particle size distribution can be drawn. The resulting frequency curves showing the volume % as a function of particle size are shown in d–f, corresponding to pictures a–c. According to TEM observations, the powder has particle sizes ranging from 20 to 100 nm. The SEM pictures reveal some agglomeration with a broad size distribution with particles of up to 2 μm and a mode value around 0.5 μm. In the case of the commercial powder, the size and morphology observed by SEM (Figure 5c) seem more homogeneous and the size distribution presents a nearly Gaussian curve with some large agglomerates of up to 2–3 μm. The size estimated by TEM gives an idea of the size of the unitary particles, but they agglomerate when the concentration increases, as happens in SEM sampling, and even more when they are in bulk or in a slurry. Accordingly, the particle size distribution was also measured for the synthesized powder treated at 800 °C (g) and the commercial untreated powder (h). It is worth noting that the commercial powder is already crystalline (probably because of some thermal treatment by the producer), whereas our synthesized powder achieves the desired crystalline structure at 800 °C. In spite of this, the commercial powder has a non-Gaussian distribution with sizes of up to near 20 μm and an average size of 2 μm. Laser measurements account for the true distribution, including the agglomerates formed by attractive interactions in the presence of air or water, and give a better indication of what will happen during processing and sintering. The results demonstrate that the powder obtained by the citrate route has a more uniform distribution and significantly lower sizes, which will be advantageous for the dispersion in water and for sintering.

**Figure 6:**
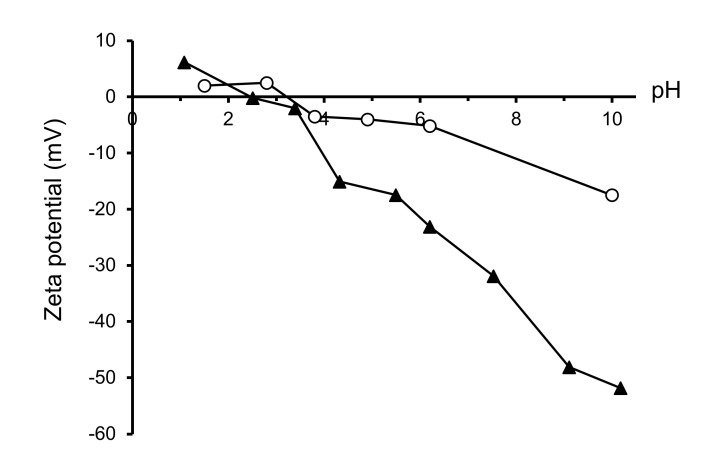
Evolution of zeta potential with pH of the synthesized and the commercial LSM powders.

The colloidal behavior in water was studied by means of zeta potential measurements. Figure 6 shows the variation of zeta potential as a function of pH for the LSM/800 °C. The zeta potential curve of the commercial LSM (28) is also shown for comparison. The zeta potential curve is very similar in both cases, with the isoelectric point occurring at pH 3.2 in the commercial powder and at pH 2.5 in the synthesized one, and very low zeta potential values at acidic pH. However, at alkaline pH, the zeta potential values are much higher for the synthesized powder, which reaches a maximum absolute value of 50 mV at pH 10, whereas the maximum absolute value for the commercial powder is around 20 mV at the same pH. This means that the stability of the synthesized powder in water is much higher, which is probably related to the lower particle size and the removal of organics after the thermal treatment that leads to pristine surfaces. It is worth noting that the commercial powder appearance suggests that the powder is spray dried and, probably, parts of the organics used in this process are still present. Other authors have reported values of ~4 [27] and ~7 [34] for the isoelectric point of La_x_Sr_1-x_MnO_3_ powders produced by combustion method and calcined at 1300 °C and 1350 °C, respectively.

**Figure 7:**
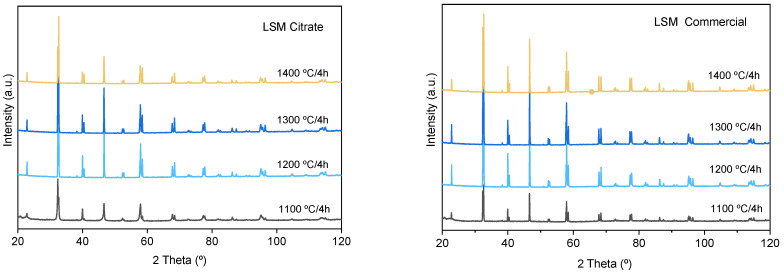
X-ray diffraction patterns of synthesized and commercial LSM compacts sintered at different temperatures.

Pressed pellets of the calcined powder were subjected to isothermal treatments of 1000–1400 °C/2 h. The XRD patterns of the calcined specimens are shown in Figure 7 in comparison with the patterns corresponding to compacts obtained with the commercial powder. In all cases, the La_0.80_Sr_0.20_MnO_3_ phase (indexed to the rhombohedral structure, space group R-3c (167), lattice parameters: a = b = 5.5227 Å, c= 13.3488 Å, α = β = 90°, γ = 120°) is the only phase detected. The calculated parameters are in good agreement with those reported by other authors for the same nominal composition of La_0.80_Sr_0.20_MnO_3_ [35,36]. The reflections of the commercial powder maintain similar intensity levels at temperatures ranging from 1200 to 1400 °C. The spectra of the compacts obtained by the citrates route show similar intensities to those obtained for the commercial one even at the different calcination temperatures up to 1300 °C, but the sample sintered at 1400 °C shows reflections with lower intensities. To understand what happens at this temperature, the densities and the microstructures were thus analyzed.

**Figure 8:**
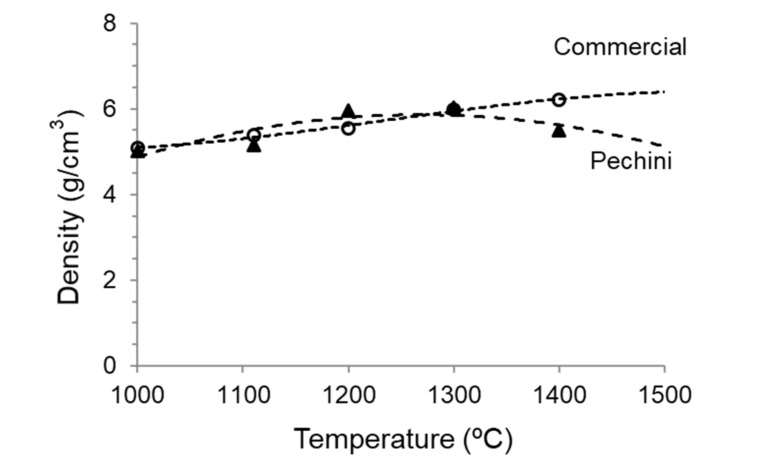
Evolution of density with sintering temperature of the compacts obtained from the synthesized and the commercial LSM powders.

The densities of the compacts measured by the Archimedes method are plotted in Figure 8, where the values of commercial powder treated at the same conditions are also shown as a reference. As can be observed, both powders have the same behavior until 1300 °C, where a density of 6.0 g/cm^3^ is achieved. An increase in the sintering temperature to 1400 °C produces a small effect in the densification of the commercial powder (up to 6.2 g/cm^3^), but, unexpectedly, leads to a slight decrease of density in the powder synthesized by the Pechini route. This can be a consequence of a grain coarsening producing higher porosity or due to chemical decomposition. This discussion can be only done in the view of the microstructural observations.

**Figure 9:**
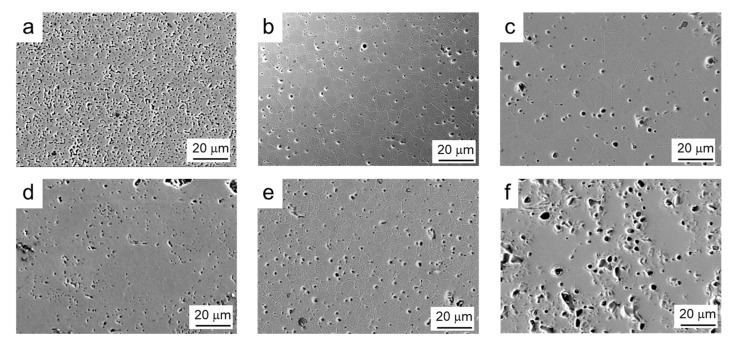
SEM microstructures of both commercial (**a**–**c**) and synthesized (**d**–**f**) powders sintered at 1200 °C (**a**,**d**), 1300 °C (**b**,**e**), and 1400 °C (**c**,**f**) with 2 h holding time.

The SEM microstructure of both commercial and synthesized powders sintered at 1200 °C, 1300 °C, and 1400 °C/2 h can be seen in Figure 9. At 1200 °C, it seems that the commercial material has a greater number of pores, but with a lower size. At 1300 °C, not only is the density similar, but also the grain size, with average values of 4–5 μm, demonstrating the quality of the synthesized powder. In both cases, pores appear at the grain boundaries and preferentially at triple points. However, in the synthesized powder treated at 1400 °C, the microstructure reveals the presence of a greater number of pores and with a larger size. This is in agreement with the measured lower density and definitely suggests some surface degradation promoted by the decomposition of manganese oxide, although its concentration is insufficient to be detected by XRD.

**Figure 10:**
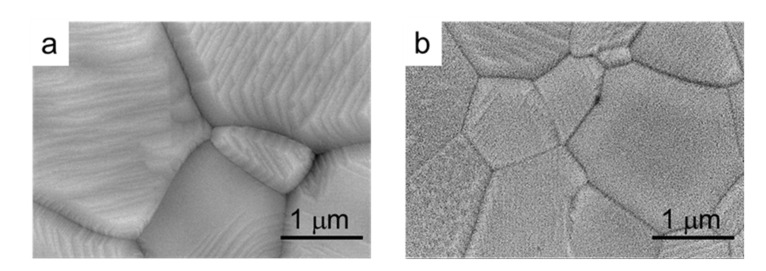
SEM microstructure of compacts of the commercial (**a**) and synthesized (**b**) LSM powders sintered at 1300 °C/2 h.

In Figure 10, high magnification pictures of compacts of both the commercial and the synthesized powders sintered at 1300 °C/2 h are shown. In these pictures, it can be observed that the microstructure of the grains is formed by layers with a high orientation, especially in the commercial powder.

### 3.2. Shaping of LSM Coatings onto YSZ Tapes

**Figure 11:**
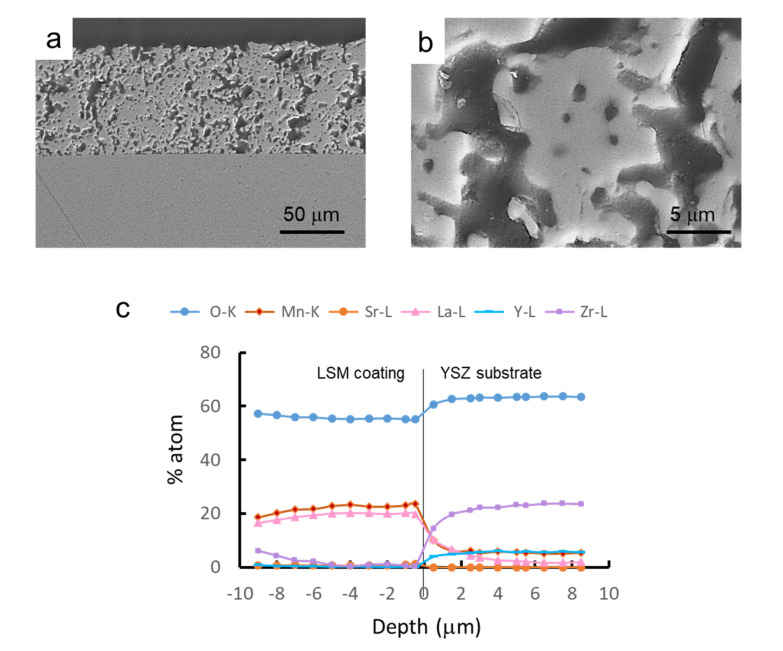
SEM picture of the cross-section of co-sintered LSM-coated YSZ (**a**), microstructure of the LSM layer (**b**), and EDS line scan profile analysis of the different elements at the interface (**c**), showing the penetration depth of the different ions from the substrate to the coating and vice-versa.

After the complete optimization of the processing and sintering conditions of the synthesized LSM powders, they were used to prepare coatings by dip coating onto YSZ substrates. In a first step, green YSZ tapes were dip coated in the LSM aqueous slurries and subsequently co-sintered at 1350 °C/2 h. Dipping tests were performed at different withdrawal rates (0.4–0.8 cm/s), with deposited mass/area values of 0.2–0.4 mg/mm^2^. Figure 11a shows a SEM picture of the cross-section of the resulting LSM-coated YSZ. It can be seen that the YSZ substrate is dense and the LSM coating is porous, as desired for application as a cathode in SOFCs. Coatings are uniform and strongly adhered to the substrate and the thickness of the coating layer is 85 ± 5 μm. In Figure 11b, a higher magnification picture of the microstructure of the LSM coating is shown. Regarding the diffusion of ions, an EDS line scan profile test was done at each side of the interface in order to determine the concentration of each element with the distance. In Figure 11c, the concentration of each atom (in %) at each side of the interface is plotted.

From the line-profile test, it can be seen that the oxygen concentration slightly decreases in the coating. The stoichiometric oxygen content values are 66 atom% and 60 atom%, very similar to those obtained by EDX, in spite of the semi-quantitative character of this tool. In the YSZ layer, Zr concentration is much higher than that of Y, as expected, and both concentrations fall at a very short distance, below 0.5 μm in the coating, demonstrating that the diffusion of the substrate elements is negligible [37] and even lower than that reported for similar materials using the same approach [38]. Nevertheless, in contact with the YSZ, Mn and La of LSM diffuse into the substrate and a cubic zirconia solid solution with divalent manganese and trivalent lanthanum ions is formed, as proposed elsewhere [39]. The thin transition layer has a total depth of by 1 μm, where the increasing concentrations of La and Mn represent a region with more intense electrochemical activity, as suggested elsewhere [38].

In the LSM side of the interface, the concentration of La ions falls progressively within a depth of 4 μm, although a very low remnant concentration remains for larger distances. Finally, Mn concentration decreases rapidly at a distance of up to 2 μm, for which a concentration of 6 atom% is reached and this concentration maintains constant for higher distances. Yokokawa reported [40] that concentrations lower than 15 atom% Mn contribute to the stabilization of the cubic structure of YSZ without precipitation of manganese oxides. An excess of Mn is often used to prevent the formation of lanthanum zirconates. This is not the case of the present work, where the Mn concentration is far below that limit and Mn diffuses into YSZ. Accordingly, the lanthanum manganite formed at the interface is depleted in Mn, thus the La activity increases, promoting its reaction with ZrO_2_, resulting in the formation of the pyrochlore phase La_2_Zr_2_O_7_ [41], detected by XRD (not shown herein). Sr diffusion is negligible and no SrZrO_3_ perovskite diffraction peaks have been observed at the interface between YSZ and LSM [42]. This is a common issue in YSZ/LSM semi-cells and has been largely reported in the literature [43,44,45,46,47,48], so that the coating produced in the present work by controlled colloidal processing is in line with the state-of-the-art.

**Figure 12:**
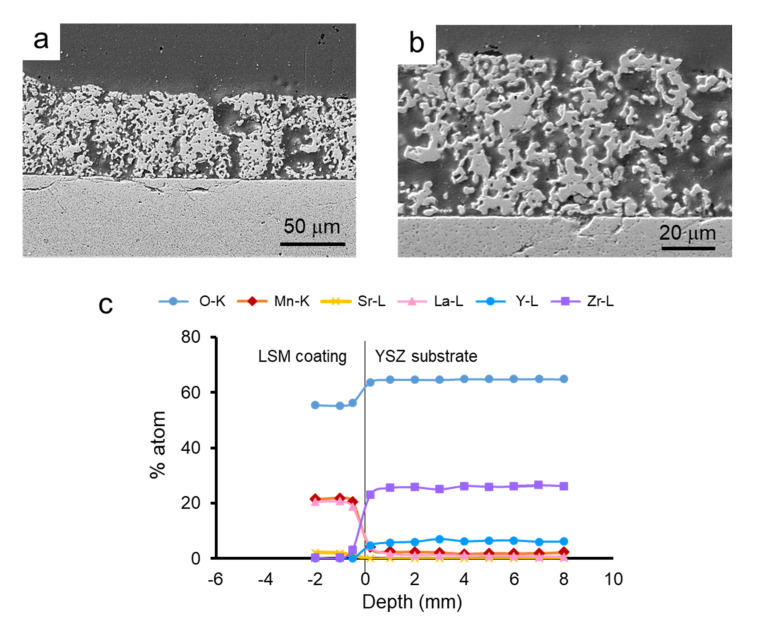
SEM picture of the cross-section LSM-coated YSZ obtained from pre-sintered tapes (**a**), microstructure of the LSM layer (**b**), and line-profile analysis of the different elements at the interface (**c**), showing the penetration depth of the different ions from the substrate to the coating and vice-versa.

Similarly, in Figure 12, the SEM microstructure of the coatings obtained by dipping the pre-sintered YSZ tapes into LSM slurries and the elemental line-profile analysis at the interface are shown. As already observed in the co-sintered coatings, the coating is porous and the substrate is dense, and the adhesion between them is very strong (Figure 12a). The coating layer is thinner than that obtained on green tapes, about 60 ± 5 μm, and the porosity is higher and more homogeneously distributed. The scan profile reveals that the diffusion of ions from one side to another of the interface is even lower than in the case of the green tape, which is expected because of the pre-sintering treatment. In the YSZ part of the interface, no diffusion of Sr can be detected. However, in the case of La and Mn, there is a very slight diffusion with a constant remaining concentration (<2%), as observed for the co-sintered materials. These values are very low and more resolution techniques should be needed to precisely determine the extent of the diffusion.

**Figure 13:**
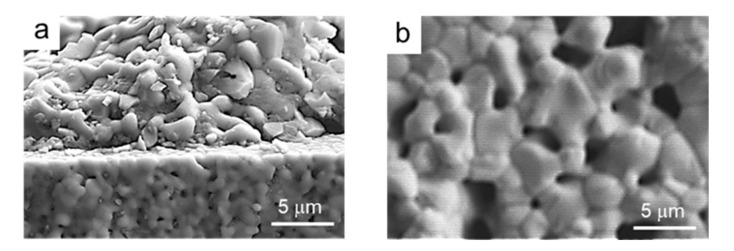
SEM microstructure of the cross section (**a**) and detail of the microstructure of the top LSM layer (**b**) of a semi cell produced using the pre-sintered tape.

Finally, Figure 13 shows the microstructure of the cross section of a semi-cell produced using the pre-sintered tape after chemical etching with HF. In the picture, the coating appears in a plane behind owing to cracking during cutting. In Figure 13b, the microstructure of the coating top surface is observed, showing the homogeneous distribution of grains and pores.

As a final consideration, it should be remarked that, although a complete analysis of the electrical properties of the materials would be required to demonstrate the advantage of the proposed approach regarding the commercial material, this being outside the scope of this paper, the proposed synthesis is very attractive for the development of SOFC semi-cells, as the resulting powder has greater colloidal stability, thus better dispersions are achieved. Consequently, more uniform microstructures can be obtained. In addition, commercial powders are hardly available and only for very few compositions, whereas this synthesis allows designing the desired relative ratios of La/Sr and at very competitive prices.

## 4. Conclusions

A strontium-doped lanthanum manganite powder with the composition La_0.80_Sr_0.20_MnO_3_ was synthesized by a citrate gel route from the corresponding metal ions nitrates using citric acid as quelating agent and PEG as poly-hydroxylated alcohol. The obtained powders were calcined at 800 °C to obtain a well crystallized powder with LSM as the unique phase. The particle size of the nanosized powder was 40–60 nm, although some agglomerates of up to 400 nm were observed. The isoelectric point occurs at pH 3.2, near that of commercial powders, but the zeta potential values are much higher (−50 mV versus −20 mV), thus demonstrating the higher stability of the synthesized powder. Densification is similar to that of the commercial powder with a maximum density at 1300 °C that tends to decrease at 1400 °C, as well as the grain size (up to 4–5 μm).

YSZ films prepared by aqueous tape casting in the green state or after pre-sintering at 1300 °C were dip-coated with 15 vol.% LSM aqueous suspensions. The use of green or pre-sintered tapes led to coating thicknesses of 85 ± 5 and 60 ± 5 μm, respectively. There is no diffusion of Zr or Y in the coating, but there is a small diffusion of La ions to short distances of 5–6 μm towards the substrate and to longer distances in the case of Mn. No diffusion is encountered for Sr. These data are in agreement with other studies of diffusion at the YSZ/LSM interface.

## Data Availability

The data presented in this study are available on request from the corresponding authors.
